# PGC-1alpha downstream transcription factors NRF-1 and TFAM are genetic modifiers of Huntington disease

**DOI:** 10.1186/1750-1326-6-32

**Published:** 2011-05-19

**Authors:** Elahe Taherzadeh-Fard, Carsten Saft, Denis A Akkad, Stefan Wieczorek, Aiden Haghikia, Andrew Chan, Jörg T Epplen, Larissa Arning

**Affiliations:** 1 Department of Human Genetics, Ruhr-University Bochum, Germany; 2 Department of Neurology, Ruhr-University Bochum, St. Josef-Hospital, Germany

## Abstract

**Background:**

Huntington disease (HD) is an inherited neurodegenerative disease caused by an abnormal expansion of a CAG repeat in the huntingtin *HTT *(*HD*) gene. The primary genetic determinant of the age at onset (AO) is the length of the *HTT *CAG repeat; however, the remaining genetic contribution to the AO of HD has largely not been elucidated. Recent studies showed that impaired functioning of the peroxisome proliferator-activated receptor *gamma *coactivator 1a (PGC-1*alpha*) contributes to mitochondrial dysfunction and appears to play an important role in HD pathogenesis. Further genetic evidence for involvement of PGC-1*alpha *in HD pathogenesis was generated by the findings that sequence variations in the *PPARGC1A *gene encoding PGC-1*alpha *exert modifying effects on the AO in HD. In this study, we hypothesised that polymorphisms in PGC-1*alpha *downstream targets might also contribute to the variation in the AO.

**Results:**

In over 400 German HD patients, polymorphisms in the nuclear respiratory factor 1 gene, *NRF-1*, and the mitochondrial transcription factor A, encoded by *TFAM *showed nominally significant association with AO of HD. When combining these results with the previously described modifiers rs7665116 in *PPARGC1A *and C7028T in the *cytochrome c oxidase subunit I *(*CO1*, mt haplogroup H) in a multivariable model, a substantial proportion of the variation in AO can be explained by the joint effect of significant modifiers and their interactions, respectively.

**Conclusions:**

These results underscore that impairment of mitochondrial function plays a critical role in the pathogenesis of HD and that upstream transcriptional activators of PGC-1*alpha *may be useful targets in the treatment of HD.

## Background

Huntington disease (HD) is an autosomal dominantly transmitted, progressive neurodegenerative disease associated with a polymorphic CAG trinucleotide repeat in the 5' part of the *HTT *(*HD*) gene, which is expanded and translated into an elongated polyglutamine tract in the huntingtin protein [1].

The length of the expanded CAG tract is inversely related to the age at clinical onset of HD, accounting for more than half of the overall variance in age at onset (AO) [2-4]. Yet, despite this strong correlation, there remains considerable variation in AO (of more than 40 years) in individuals with identical repeat lengths. Evidence has been provided for genetic as well as for environmental factors that affect the AO [5]. Identifying these modifiers in human HD and defining their precise role in the causal pathogenesis of HD could help to develop more effective treatment regimen for HD. To date, several candidate modifier genes of HD have been described in independent studies, all of them implicating a variety of processes apparently contributing to HD pathogenesis [6-10]. Recently, mitochondrial DNA (mtDNA) haplogroup H (7028C) and variations in the peroxisome proliferator-activated receptor *gamma *coactivator 1a (*PPARGC1A*) gene encoding PGC-1*alpha *were shown to exert modifying effects on the AO in HD, thus providing genetic evidence that complex interrelations of mitochondrial dysfunction have effects on the pathogenic process in HD [11-14]. A number of studies suggested that PGC-1*alpha *dysfunction may be central to HD pathogenesis [15]. PGC-1*alpha*-deficient mice show hyperkinetic movement disorder and striatal degeneration [16,17]. Gene expression analyses in cell lines, transgenic mouse models of HD and in different tissues from HD patients revealed a disruption of the PGC-1*alpha *regulatory pathway [18-20]. Recently, investigations on the ability of AMP-activated protein kinase (AMPK) to activate PGC-1*alpha *in brain, liver, brown adipose tissue (BAT) and muscle of HD transgenic mice strengthened the theory that impaired activation of PGC-1*alpha *plays an important role in the metabolic disturbances involved in the pathology of HD [20,21]. Accordingly, modulation of PGC-1*alpha *levels and activity has been proposed as a therapeutic option for HD pathology [15]. Indeed, activation of the PGC-1*alpha *signaling pathway via resveratrol-induced activation of the silent information regulator T1 (SIRT1), a mammalian sirtuin, in transgenic mice achieved positive effects in BAT [22]. Yet, PGC-1*alpha *as a coactivator protein responds to environmental influences and subsequently regulates various pathways in a tissue-specific and highly coordinated manner. Therefore, such pharmacological interventions aimed at PGC-1*alpha *may suffer from lack of specificity. Targeting key factors of the wide PGC-1*alpha *transcriptional network could, therefore, represent another approach for a specific modulation of PGC-1*alpha *activity. PGC-1*alpha *controls many aspects of oxidative metabolism, including respiration and mitochondrial biogenesis through coactivation and enhancing the expression and activity of several transcription factors including the nuclear respiratory factors (NRF)-1 and NRF-2 (GABP) and the estrogen related receptor alpha (ERR*alpha*) [23,24]. PGC-1*alpha *is also indirectly involved in regulating the expression of mtDNA transcription via increased expression of mitochondrial transcription factor A (TFAM) which is coactivated by NRF-1 [23,25]. In the present study, we addressed the question of the role of a diverse set of PGC-1*alpha *related factors in modifying the AO of HD. We investigated polymorphisms in the genes encoding ERR*alpha *(*ESRRA*), Mitofusin 2 (*MFN2*), NRF-1 and NRF-2 (*NRF-1*, *GABPA *and *GABPB1*), PGC-1*beta *(*PPARGC1B*), peroxisome proliferator-activated receptor-*gamma *(*PPARG*), SIRT1 (*SIRT1*) and TFAM (*TFAM*, see Table 1). A more comprehensive understanding of the pleiotropic effects of the PGC-1*alpha *family regulatory network in mitochondrial biogenesis and HD pathogensis could help to identify and fine tune pharmacological interventions targeting PGC-1*alpha *or alternatively its transcriptional complexes.

**Table 1:** Candidate gene and SNP characteristics

Gene		Chromosome	SNPs	Selection Criteria
*ESRRA*		11q13.1	rs3217060	23 bp microsatellite repeat located in promoter region

*MFN2*		1p36.22	rs3753579	located in promoter region

*NRF-1*		7q32.2	rs7781972, rs6949152	previously reported association [29]
			
			rs1882094, rs3735006,	non-synonymous SNPs
			
			rs10275661, rs10225103, rs10268267, rs6962005, rs10231985, rs11487138, rs11761434, rs1962039, rs2402970, rs6948697, rs10500120	tag SNPs using public databases (dbSNP)

*NRF-2*	*GABPA*	21q21.3	rs2829897 (A291V), rs2829898 (W323X), rs2829900 (E345K)	non-synonymous SNPs
	
	*GABPB1*	15q21.2	rs12594956, rs8031031	previously reported association [39]

*PPARGC1B*		5q33.1	rs7732671 (A164P), rs11959820 (R253S)	non-synonymous SNPs

*PPARG*		3p25.2	rs1801282 (P12A)	non-synonymous SNP
			
			rs2938392, rs3856806	tag SNPs using public databases (dbSNP)

*SIRT1*		10q21.3	rs3758391, rs7069102	previously reported association [40]
			
			rs10997860, rs2273773, rs35461348	tag SNPs using public databases (dbSNP)

*TFAM*		10q21.1	rs1937 (S12T)	non-synonymous SNP
			
			rs4390300, rs10826178, rs1049432, rs11006132	tag SNPs using public databases (dbSNP)

## Results

**Figure 1:**
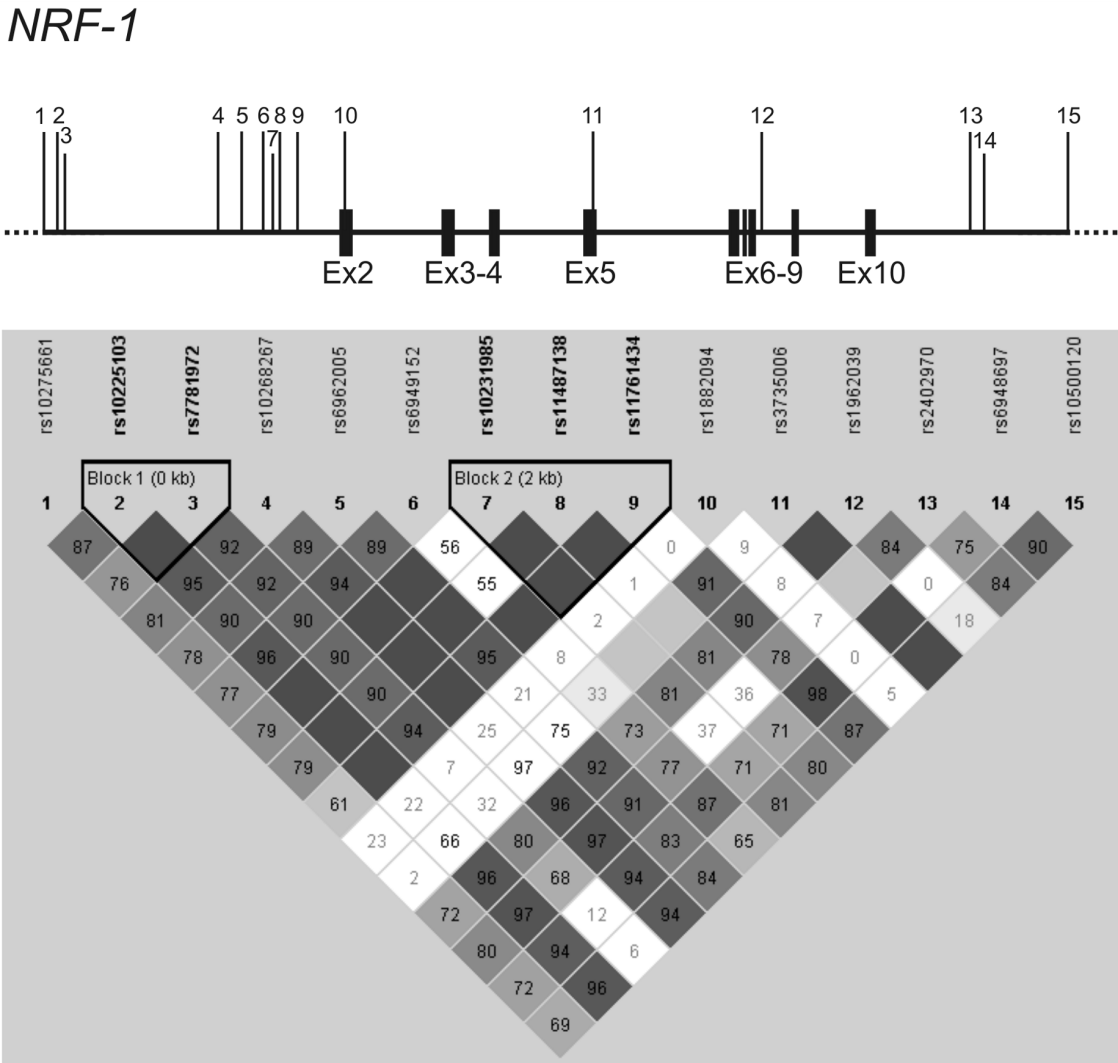
**Graphical representation of single-nucleotide polymorphisms (SNPs) in relation to the exon-intron structure (top) and the Haploview pairwise linkage disequilibrium (LD) structure of part of *NRF-1 *(bottom)**. Exons are indicated by solid black boxes, and the numbered vertical lines indicate positions of the SNPs analysed in *NRF-1*. Haploview plot showing pairwise LD (D' values) for all 15 SNPs based on genotypes of 401 HD patients of the study. Each square plots the level of LD between a pair of SNPs, comparisons between neighboring SNPs are arranged along the first line under the names of the SNPs. Dark grey coloring indicates strong LD, medium grey shading indicates less strong LD, light grey indicates intermediate LD, and white indicates weak LD. LD blocks are framed in black and were classified according to Gabriel et al. (2002).

In our cohort of 401 HD patients, the expanded *HTT *allele accounts for nearly 73% of the variance in motor AO (R^2 ^= 0.729) and shows a highly significant influence on the AO (p < 0.0001). Multiple regression models were used to test all SNPs for association with the AO. Of these, four showed a nominal p-value < 0.05 (Table 2). For *NRF-1*, addition of the intronic variations rs7781972 and rs6949152 showed an association with the motor AO. Inclusion of the rs7781972 genotypes in the model increased the R^2 ^statistic from 0.729 to 0.733 in both the dominant and the additive model (p = 0.017 and p = 0.011, Table 2). Inclusion of the rs6949152 genotypes increased the R^2 ^from 0.729 to 0.734 (p = 0.004) according to the dominant model and to 0.733 (p = 0.013) in the additive model (Table 2). Examining linkage disequilibrium (LD) among the 15 *NRF-1 *variations revealed, that the variations rs10275661, rs10225103, rs7781972, rs10268267, rs6962005 and rs6949152 in IVS1 were in high LD in the cohort (*D*' = 1.0, *r*^2 ^= 0.87-0.92). In 3' direction the LD breaks down, and a second block of very strong LD (*D*' = 1.0, *r*^2 ^≥ 0.98) is observed for rs10231985, rs11487138 and rs11761434. The remaining SNPs covering exon 2 to IVS10 (rs1882094, rs3735006, rs1962039, rs2402970, rs6948697 and rs10500120) showed lower LD coefficients (D') and r^2 ^values (Figure 1).

**Table 2:** Details of SNPs included in the multivariable model

Gene	SNP	Chromosome	**Allele Freq**.	Type	Biological Processes	**ΔR**^**2**^	Standardized beta		
							
							Coefficients	t	P *
*NRF-1*	rs7781972	7q32.2	189/170/42	IVS1	control of nuclear genes required for respiration, heme biosynthesis, and mtDNA transcription and replication	.004	-.066	-2.56	.011
									
	rs6949152		289/101/11	IVS1		.005	-.076	-2.94	.004

*TFAM*	rs1049432	10q21.1	239/145/17	3' near gene	mtDNA transcription and maintenance factor	.002	-.053	-2.04	.042
					
	rs11006132		208/170/23	3' near gene		.004	-.063	-2.45	.015

*PPARGC1A*	rs7665116	4p15.2	309/82/10	IVS2	transcriptional coactivator, regulation of key mitochondrial genes	.004	-.065	-2.52	.012

*CO1*	C7028T	mt7028	207/194	mitochondrial	component of the respiratory chain, catalyses the reduction of oxygen to	.003	-.057	-2.22	.027

The variability in motoric AO attributable to the CAG repeat length was assessed by linear regression using the logarithmically transformed onset age as the dependent variable and genotypes as independent variables. Delta (**Δ**) R^**2 **^quantifies the relative improvement of the regression model when the genotypes are considered in addition to the CAG repeats. * nominal *P*-values (not adjusted for multiple testing)

In *TFAM *two SNPs showed association with motor AO among which, rs11006132 in the 3' region of the gene was most strongly associated (0.729 to 0.733; p = 0.015, Table 2). Including the genotypes of the coding Ser12Thr polymorphism (rs1937) in the model for AO did not increase the R^2 ^statistic. The *TFAM *variations were in moderate LD with one another (*D*' < 1.0, *r*^2 ^= 0.34-0.47), only the associated variations rs1049432 and rs11006132 in the 3' region are highly correlated with each other (pairwise *r*^2 ^values >0.97). All other selected polymorphisms in *NRF2*, *SIRT1*, *PGC1beta*, *MFN2 *and *PPARgamma *showed no significant influence on the AO.

After consideration of SNP genotypes individually, a multivariable model was built in order to determine if a significant proportion of the variation in AO could be explained by the joint effect of mitochondrion-related modifier variations and their interactions. *NRF-1 *and *TFAM *SNPs showing significant main effects (nominal *P *< 0.05) together with the previously analysed modifier variations in *PPARGC1A *(rs7665116) and *CO1 *(C7028T, defining mt haplogroup H) were included in a multivariable model (Table 2). Here, the main effects together with all possible pairwise interactions of the SNPs were included in a forward selection process. The final multivariable model increased the R^2 ^statistic from 0.729 to 0.747 and explained 4.8% additional residual variance in the motor AO of HD (Table 3).

**Table 3:** SNP-SNP interaction included in final multivariable model

Interaction	**R**^**2**^	**Adjusted R**^**2**^	**ΔR**^**2**^	Standardized beta		
				
				Coefficients	t	*P*
CAG	.730	.729		-.864	-34.23	<0.0001
*NRF-1 *rs6949152 * *CO1 *C7028T	.739	.738	.009	-.090	-3.55	<0.0001
*NRF-1 *rs7781972 * *TFAM *rs11006132	.745	.743	.014	-.077	-3.03	<0.003
*PPARGC1A *rs7665116	.749	.747	.018	-.069	-2.72	<0.007

A multivariable model including a total of four interactions was built in the total HD cohort using forward selection. With the addition of each SNP-SNP interaction, along with each SNP's respective main effect, the variability in adjusted log motor AO increased (assessed by adjusted R^2^). The final multivariable model explains 4.8% additional residual variance in HD motor AO.

**Figure 2:**
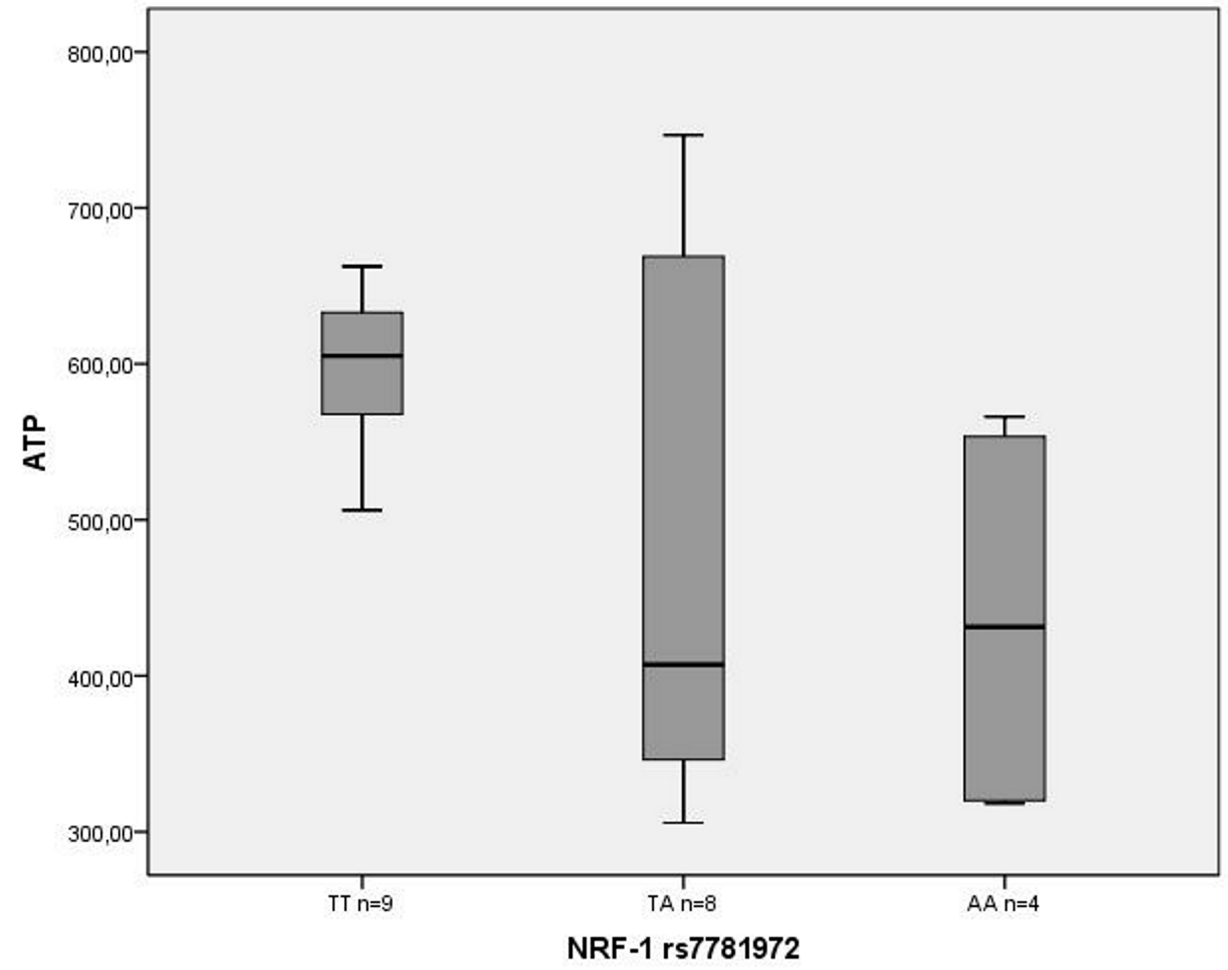
**ATP concentration in HD patients**. Box plot shows medians, quartiles and extreme values. The mean ATP levels of HD patients with the TT genotype (600.6 ± 48.7 ng, n = 9) and those carrying at least one rare *NRF-1 *rs7781972 allele (TA and AA, 487.1 ± 179 ng, n = 8; 436.7 ± 135.1 ng, n = 4) were significantly different (p = 0.03 two-sample *t *test).

When correlating the ATP concentrations with the *NRF-1 *and *TFAM *genotypes, HD patients carrying at least one rare *NRF-1 *rs7781972 allele showed significantly lower ATP concentrations (487.1 ± 179 ng, n = 8; 436.7 ± 135.1 ng, n = 4) than homozygous individuals carrying the frequent allele (600.6 ± 48.7 ng, n = 9, p = 0.03; Figure 2). Considering an additive allele effect the ATP levels were negatively correlated with the rare *NRF-1 *rs7781972 allele (Pearson coefficient -0.478, p < 0.029). Yet, this effect was not obvious in a group of 38 healthy controls (529 ± 175.5 ng, n = 14; 487.4 ± 148 ng, n = 22 *vs*. 492 ± 120.5 ng, n = 2, Pearson coefficient -0.130, p < 0.437). In both groups the ATP levels were not significantly correlated with the mtDNA:nDNA ratios (HD: Spearman coefficient -0.383, p < 0.095, controls: 0.134, p < 0.417). Regarding the entire patient cohort (n = 401), the mtDNA content was also not associated with age, sex, AO, disease duration, CAG repeat lengths or any other genotype.

## Discussion

Here, we performed an association study for AO modifiers of HD, based on a candidate gene approach including variations in PGC-1*alpha *target genes involved in the regulatory network that controls mitochondrial function. We found that SNPs in *NRF-1 *and *TFAM *showed nominally significant association with AO of HD.

The NRFs and - most importantly NRF-1 - are potent stimulators of the expression of nuclear genes required for mitochondrial respiratory function [23-25]. NRF-1 directly regulates the expression of several nuclear encoded genes involved in the expression, assembly and functions of the respiratory chain or indirectly regulates the mitochondrion-encoded cytochrome c oxidase (COX) subunit genes by activating TFAM [23-25]. Beyond the transcriptional expression of the respiratory chain NRF-1 is also supposed to control the key components of the protein import and assembly machinery, thus suggesting a broader meaning for NRF-1 in orchestrating events in the mitochondrial biogenesis [26]. Yet, a direct functional effect of the associated polymorphisms remains to be determined. Comparing the mtDNA content with various clinical and genetic parameters of the HD patients did not yield statistically significant results. Yet, the interpretation of these results has to be considered with caution, since expressing the mitochondrial DNA concentration as a ratio to nuclear DNA varies dependent on blood-processing protocols [27].

Given the relatively small sample sizes, the additive allele effect of *NRF-1 *rs7781972 on the ATP levels in HD patients should be regarded as preliminary. Nevertheless, these data could be indicative of genotype-dependent variation in the response to chronic energy stress conditions. Since high-intensity exercise also causes metabolic stress, physical activity on a very high level for prolonged time implies, amongst other effects, increased oxidative stress and the consequences of cellular dysfunction due to insufficient supply of ATP [28]. In this context, it is interesting to note that *NFR-1 *genotypes - particularly regarding rs6949152 - significantly influence phenotype traits indicative of endurance capacity in humans and their trainability [29].

Deviation in the response of certain *NRF-1 *genotypes on chronic energy stress conditions (endurance exercise training, but also neurodegeneration) could therefore explain the association with the variation in the onset age of HD. The same could apply to the other genotypes, since *TFAM*, *PPARGC1A *and mt haplogroups have also been described to be associated with differences in physical capabilities and training-induced effects [30-34]. Recently, Chaturvedi et al. [21] demonstrated that chronic energy deprivation in mice by administering the catabolic stressor β-guanidinopropionic acid increased expression of PGC-1*alpha*, NRF-1 and TFAM. Yet, this pathway, leading to mitochondrial biogenesis, increased mtDNA and numbers of mitochondria in response to energetic stress, was blocked in HD transgenic mice [21]. Furthermore, when combining the newly detected modifier variations in *NRF-1 *and *TFAM *with the previously described modifier variations in *PPARGC1A *and mt haplogroup H, in our study much more variability in AO can be explained than in seperate analyses. The combination of the polymorphisms defines nearly 5% of the unexplained variance in residual AO in our sample, thus highlighting their coordinately regulated metabolic interplay and the possible involvement in pathogenic HD conditions.

The important role of PGC-1*alpha *in the regulation of mitochondrial function together with the association between mitochondrial dysfunction and HD pathogenesis implies that activation of PGC-1*alpha *could have critical potential in the treatment of HD. Yet, pharmacological interventions directly aimed at PGC-1*alpha *have to overcome inherent limitations of targeting a coactivator protein [35]. Therefore, targeting the regulators of PGC-1*alpha*, as already demonstrated for its down-stream target ERR*alpha *and the NAD^+^-dependent deacetylase SIRT1, may represent an alternative approach [36].

## Conclusions

Polymorphisms in *NRF-1 *and *TFAM *influence the AO of HD. Furthermore, we have demonstrated evidence for gene-gene (SNP-SNP) interactions among these SNPs and the modifier variations in *PPARGC1A *and *CO1*, thus providing further genetic evidence that impaired mitochondrial biogenesis in response to energetic stress plays a critical role in the pathogenesis of HD. These data also support the idea that upstream transcriptional activators of PGC-1*alpha *may be useful in the treatment of HD. However, since no multiple testing correction was applied, caution is necessary in interpreting. Further studies will be necessary to replicate these associations and to elucidate the pathways through which the modifier variations exert their effects on metabolic deficits underlying HD pathogenesis and potentially other late-onset diseases.

## Methods

### Study population

The study population has been described before [12] and consisted of 401 unrelated German patients (208 men and 193 women) with the clinical and genetic diagnosis of HD, recruited from the Huntington Center NRW, Bochum (Germany). *AO was defined as the age at which, according to the experienced neurologists of the Center, the first motor signs of HD appeared (motor AO). The expanded trinucleotide repeats ranged from 40 to 66 with a *mean (*±SD) of 44.48 ± 3.8 *CAGs, motor *AO ranged from 16 to 76 years, with an onset (*mean *± SD) of 44.9 ± 11.6 *years. *The normal CAG blocks ranged from from 10 to 32 with a *mean (*±SD) of 18.24 ± 2.8 *CAGs. *HD CAG repeat sizes were determined by polymerase chain reaction using an assay counting the perfectly repeated (CAG)*_*n *_*units*. The study was performed in a manner that fully complies with the Code of Ethics of the World Medical Association (Declaration of Helsinki) and was approved by the ethics review board of the Ruhr-University Bochum (Germany).

### Candiate gene and SNP selection strategy

In order to estimate a possible modifier effect conferred by individual SNPs, as well SNP-SNP interactions, we studied SNPs from 9 candidate genes contributing to the PGC-1*alpha *family regulatory network in mitochondrial biogenesis (Table 1). The candidate genes and SNPs were selected using the available published evidence at the beginning of the project. SNPs were chosen based on a number of different criteria including the published data, non-synonymous SNPs and tag SNPs from public databases such as dbSNP [http://www.ncbi.nlm.nih.gov/SNP].

### Genotyping

We studied a total of 15 SNPs in *NRF-1 *(rs10275661, rs10225103, rs7781972, rs10268267, rs6962005, rs6949152, rs10231985, rs11487138, rs11761434, rs1882094, rs3735006, rs1962039, rs2402970, rs10500120, rs6948697), three in *GABPA *(*NRF*-2a, rs2829897, rs2829898, rs2829900), two in *GABPB1 *(*NRF*-2b, rs12594956, rs8031031), five in *TFAM *(rs4390300, rs1937, rs10826178, rs1049432, rs11006132), five SNPs in *SIRT1 *(rs3758391, rs10997860, rs7069102, rs2273773, rs35461348), two in *PPARGC1B *(rs11959820, rs7732671), one in *MFN2 *(rs3753579), three in *PPARγ *(rs1801282, rs2938392, rs2938392) and one in *ESRRA *(rs3217060). Genotyping was performed by PCR-RFLP techniques and commercially available TaqMan genotyping assay (Applied Biosystems). For fragment analysis of the 23 bp microsatellite repeat (ESRRA23, rs3217060) in the promoter of *ESRRA *and the insertion-deletion (*INDEL*) polymorphism (rs35461348) in *SIRT1 *we used fluorescence 5'FAM labelled, tailed oligonucleotide added to the 5'-part of the sequence specific primer as described before [37]. All primers were designed with the Primer Express 2.0 Software (Applied Biosystems, Foster City, USA). All other details of the methodology and primer sequences are available upon request.

### mtDNA Quantification

Quantitative real-time PCR (qPCR) was used for mitochondrial DNA content measurement using an Applied Biosystem StepOne cycler (Applied Biosystems, Foster City, CA). Correction for mtDNA quantity was performed by simultaneous measurement of a single copy nuclear RNAseP gene. Quantification of nuclear (n) DNA was done with a commercial kit (RNAseP, Control Reagents, Applied Biosystems P/N 4316844) together with nDNA-specific fluorescent probe which was labelled internally using VIC fluorescent dye. Two primers and one probe used for mtDNA 12S ribosomal RNA quantification which were as here: mtF805 (5'CCACGGGAAACAGCAGTGATT3'), mtR927 (5'CTATTGACTTGGGTTAATCGTGTGA3') and TaqMan probe (Applied Biosystems) (6FAM-5'TGCCAGCCACCGCG3'-MGB) (labelled at the 5' end with a fluorescent reporter, 6FAM). The 10-μL PCR reaction contains 1 × TaqMan Universal PCR Master Mix (Applied Biosystems P/N 4304437), 0.5 μL of PDARs RNAseP and 112 nM of each mtDNA primer, 125 nM of mtDNA TaqMan probe, and 25 ng of total genomic DNA extract. PCR conditions were 2 min at 50°C and 10 min at 95°C, followed by 40 cycles of 15 sec of denaturation at 95°C and 60 sec of annealing/extension at 60°C.

In order to determine the quantities of mtDNA and nDNA, the average threshold cycle number (Ct) values of the nDNA and mtDNA were obtained from each case. Measurements were performed in triplicates and presented as means. The level of mtDNA was calculated using the delta Ct (ΔCt) of average Ct of mtDNA and nDNA (ΔCt = CtmtDNA-CtnDNA) in the same well as an exponent of 2 (2ΔCt). To test reproducibility a constant reference sample was analysed in each run.

### Assessment of intracellular ATP concentrations in peripheral leukocytes

Sodium heparin blood was obtained from 21 HD patients and 38 age-/sex matched healthy controls devoid of acute infections (clinical aspect, white blood cell count, C-reactive protein). Assessment of intracellular ATP concentrations was performed as described before [13].

### Statistical analysis

Variability in AO attributable to the CAG repeat number was controlled by linear regression using the logarithmically transformed AO as the dependent variable SNP genotypes as independent variables. All analyses were performed assuming a dominant or an additive effect for each polymorphism. In the dominant model, both, the heterozygous and the rarely observed homozygous variation were combined. In the additive model, both, rare homozygous and heterozygous variation effects were estimated using two dummy variables. We used a two-stage approach in order to identify both main and interactive genetic effects associated with the motor AO. In the first stage we conducted association analyses for SNP main effects. After this, we performed multivariable SNP modelling with associations passing the first stage of analysis as well as two previously published modifier SNPs in PPARGC1A (rs7665116) and CO1 (C7028T) [12,13]. The results were not adjusted for multiple testing as a Bonferroni adjustment would have been very conservative when taking into account that the SNPs were not independent, rather they were in tight LD. Hardy-Weinberg equilibrium (HWE) was tested for each SNP. Relationships between variables were determined by Pearson's correlation coefficient. The strength of LD between pairs of SNPs was measured as D' by using HAPLOVIEW [http://www.broad.mit.edu/mpg/haploview/]. LD blocks were inferred from the definition proposed by Gabriel et al. [38] as implemented in HAPLOVIEW with D' confidence bounds of 0.7-0.92. Comparison of dependent variables was performed using unpaired t tests with nominal significance assigned when p ≤ 0.05. SPSS Ver.18.0 (SPSS Inc.) was used for all statistical analyses.

## Competing interests

The authors declare that they have no competing interests.

## Authors' contributions

ETF carried out the molecular genetic experiments. CS ascertained the clinical status of the patients. DAA, SW, AH and AC contributed to the experimental design. JTE supervised the overall project, and LA designed the study including statistical analysis and drafted the manuscript. All authors read and approved the final version of the manuscript.
